# Extraction Optimization, Structural Characterization, and Anti-Hepatoma Activity of Acidic Polysaccharides From *Scutellaria barbata* D. Don

**DOI:** 10.3389/fphar.2022.827782

**Published:** 2022-04-04

**Authors:** Wenwen Su, Leilei Wu, Qichao Liang, Xiaoyue Lin, Xiaoyi Xu, Shikai Yu, Yitong Lin, Jiadong Zhou, Yang Fu, Xiaoyan Gao, Bo Zhang, Li Li, Dan Li, Yongkui Yin, Gaochen Song

**Affiliations:** ^1^ Department of Biochemistry and Molecular Biology, Mudanjiang Medical University, Mudanjiang, China; ^2^ Collage of Pharmacology, Mudanjiang Medical University, Mudanjiang, China; ^3^ The First Clinical College of Medicine, Heilongjiang University of Chinese Medicine, Harbin, China; ^4^ The First Clinical College of Medicine, Mudanjiang Medical University, Mudanjiang, China; ^5^ Collage of Basic Medicine, Heilongjiang University of Chinese Medicine, Harbin, China; ^6^ Collage of Basic Medicine, Mudanjiang Medical University, Mudanjiang, China; ^7^ Department of Oncology, Mudanjiang Cancer Hospital, Mudanjiang, China; ^8^ College of Life Sciences, Mudanjiang Medical University, Mudanjiang, China

**Keywords:** *Scutellaria barbata* D. Don, polysaccharides, purification, structure, anti-hepatoma activity

## Abstract

The Chinese medicinal herb *Scutellaria barbata* D. Don has antitumour effects and is used to treat liver cancer in the clinic. *S. barbata* polysaccharide (SBP), one of the main active components extracted from *S. barbata* D. Don, exhibits antitumour activity. However, there is still a lack of research on the extraction optimization, structural characterization, and anti-hepatoma activity of acidic polysaccharides from *S. barbata* D. Don. In this study, the optimal extraction conditions for SBP were determined by response surface methodology (RSM): the material-liquid ratio was 1:25, the extraction time was 2 h, and the extraction temperature was 90°C. Under these conditions, the average extraction efficiency was 3.85 ± 0.13%. Two water-soluble polysaccharides were isolated from *S. barbata* D. Don, namely, SBP-1A and SBP-2A, these homogeneous acidic polysaccharide components with average molecular weights of 1.15 × 10^5^ Da and 1.4 × 10^5^ Da, respectively, were obtained at high purity. The results showed that the monosaccharide constituents of the two components were fucose, galactosamine hydrochloride, rhamnose, arabinose, glucosamine hydrochloride, galactose, glucose, xylose, and mannose; the molar ratio of these constituents in SBP-1A was 0.6:0.3:0.6:30.6:3.3:38.4:16.1:8:1.4, and that in SBP-2A was 0.6:0.5:0.8:36.3:4.4:42.7:9.2:3.6:0.7. In addition, SBP-1A and SBP-2A contained uronic acid and *β*-glucan, and the residue on the polysaccharide was mainly pyranose. The *in vitro* results showed that the anti-hepatoma activity of SBP-2A was better than that of SBP-1A and SBP. In addition, SBP-2A significantly enhanced HepG2 cell death, as cell viability was decreased, and SBP-2A induced HepG2 cell apoptosis and blocked the G1 phase. This phenomenon was coupled with the upregulated expression of P53 and Bax/Bcl-2 ratio, as well as the downregulated expression of the cell cycle-regulating protein cyclinD1, CDK4, and Bcl-2 in this study. Further analysis showed that 50 mg/kg SBP-2A inhibited the tumour growth in H22 tumour-bearing mice, with an average inhibition rate of 40.33%. Taken together, SBP-2A, isolated and purified from *S. barbata* showed good antitumour activity *in vivo* and *in vitro*, and SBP-2A may be a candidate drug for further evaluation in cancer prevention. This study provides insight for further research on the molecular mechanism of the anti-hepatoma activity of *S. barbata* polysaccharide.

## Background

Hepatocellular carcinoma (HCC) is the fourth leading cause of cancer-related death and ranks sixth among new cases of cancer worldwide (Yang et al., 2019). Because of the lack of symptoms at early stages and high heterogeneity of liver cancer, most patients are diagnosed in the late stages, resulting in a low cure rate. At the current rate, the number of new cases and deaths due to liver cancer worldwide are expected to increase from 841,080 to 781,631 in 2018–1,361,836 to 1,284,252 in 2040, which are increase of 62 and 64%, respectively (Singal et al., 2020). The methods for treating liver cancer include surgical resection, liver transplantation, arterial chemoembolization, and systemic pharmacological treatment with the kinase inhibitor sorafenib. However, nearly 70% of patients easily develop recurrent liver cancer or metachronous liver cancer (Tabrizian et al., 2015), with typical side effects such as hypertension, hypothyroidism, and leucopenia/neutropenia (Finn et al., 2020). Although the worldwide incidence and mortality rates of liver cancer are increasing compared with the burdens of many other cancers, the development of new drugs for liver cancer has been lacking historically. Therefore, developing low toxicity and high-efficiency antitumor drugs has become a research focus.

Polysaccharides are among the main bioactive substances of Chinese herbal medicine and have a wide range of pharmacological effects, including antitumor effects (Gan et al., 2020), antioxidant effects (Wei et al., 2015), anticoagulant effects (Zhao et al., 2012), effects in the treatment of ischemia-reperfusion injury (Yuan et al., 2021), liver protection effects (Qu et al., 2020), antiviral effects (Cao et al., 2020), effects in the control of blood lipids (Zhang et al., 2014), effects in the control of blood pressure (Xie et al., 2021) and immunoregulatory effects (Wang et al., 2021). *Scutellaria barbata* D. Don is a plant of the family Lamiaceae (Yeon et al., 2015), and is listed in the 2015 edition of the Chinese Pharmacopoeia. It has the characteristic effects of clearing away heat, detoxifying, diuresis and detumescence. *S. barbata* polysaccharide (SBP) exhibits anticomplement activity (Wu and Chen, 2009), inhibits lung cancer growth (Yang et al., 2014; Yang et al., 2015), and inhibits the proliferation and metastasis of colorectal cancer HT29 cells (Sun et al., 2017; Li H. et al., 2019) and high glucose-induced retinal vascular endothelial cells (Li and Xiao, 2021). However, most studies on SBP have examined crude polysaccharides, while the isolation and purification of SBP have not been performed, and its anti-hepatoma activity has not yet been studied. Furthermore, the lack of clear structural and mechanistic information limits the clinical application of the unique antitumour properties of SBP. *S.barbata* has been widely used in the treatment of digestive system tumours, especially liver cancer. In addition, our previous study showed that SBP inhibited tumour growth in H22 hepatoma-bearing mice (Li L. et al., 2019). However, there is still a lack of research on the effect of SBP on liver cancer and the underlying mechanism. Therefore, HCC cells were selected as the tumour target for this research.

Based on the above considerations, this study used water extraction and alcohol precipitation to extract crude polysaccharides from *S. barbata*, response surface methodology (RSM) to optimize the extraction process and column chromatography to purify SBP. We preliminarily identified the polysaccharides content, molecular weight, monosaccharide composition and basic structure of *S. barbata*. The anti-hepatoma activity and mechanism of SBP were further studied. We hope that this study will provide a basis for elucidating the antitumour effects of *Scutellaria barbata* polysaccharides.

## Materials and Methods

### Materials

*S. barbata* was purchased from Hebei Jinyezi Pharmaceutical Co., Ltd. (Hebei, China); the finely sieved powder was dried to a constant weight. The fine powder was crushed through a 40 mesh sieve and stored in the dryer until use. DEAE-52 cellulose was purchased from Phygene Biotechnology Co., Ltd. (Fuzhou, China), and Sephadex G-100 was purchased from GE Healthcare Bio-Sciences AB (Uppsala, Sweden). Monosaccharide standards were provided by Shenzhen Bo Rui Sugar Biology Co., Ltd. (Shenzhen, China). All other reagents used in the tests were analytical grade and obtained from local chemical suppliers in China. The HepG2 cell line was purchased from the Chinese Academy of Sciences (Shanghai, China). Dry MEM powder, penicillin and streptomycin, trypsin, nonessential amino acids (NEAAs), and sodium pyruvate in a solution containing 100 μg/ml penicillin and 100 μg/ml streptomycin were purchased from GIBCO (Grand island biological company, NY, USA). The H22 cell line and Australian foetal bovine serum (FBS) were purchased from Wuxi Xinrun Biotechnology Co., Ltd. (Wuxi, China). 3-(4,5-Dimethylthiazol-2-yl)-2,5-diphenyltetrazolium bromide (MTT) cell proliferation and cytotoxicity test kits and DNA content test kits were purchased from Solebao (Beijing, China). A Hoechst 33258 apoptosis staining kit was purchased from Beyotime Institute of Biotechnology. (Nanjing, China).

### Optimization of Water Extraction and Alcohol Precipitation With RSM

#### Single-Factor Experimental Design

Chunlin Ye used RSM to optimize the extraction parameters for *S. barbata*, and the polysaccharide extraction rate was only 2.43 ± 0.11% (Ye and Huang, 2012). To improve the crude polysaccharide extraction rate for *S. barbata*, we selected three factors: material-liquid ratio, extraction temperature and extraction time. Five levels were studied for each factor (Zhao et al., 2015; Qu et al., 2016), including material-liquid ratios of 1:10, 1:15, 1:20, 1:25, and 1:30; extraction temperatures of 60, 70, 80, 90, and 100°C; and extraction time of 1, 2, 3, 4, and 5 h. The extraction rate was calculated from the measured SBP content.

#### RSM

According to the results of the single-factor experiments, the three factors and five levels were applied in a Box-Behnken experimental design to optimize the extraction conditions. The relevant experiments were performed by Design Expert 11 (State-Ease Inc., Minneapolis, MN, United States) RSM software and repeated three times for each group. RSM was used to perform regression analysis on the experimental data, and a nonlinear quadratic model was fitted according to the formula (Wang et al., 2012; Jia et al., 2014).

### Extraction of SBP by Water Extraction and Alcohol Precipitation

*S. barbata* powder was soaked several times in 95% ethanol solution to decolorize the powder and remove alcohol-soluble components. The residue was dried for 24 h to prepare the *S. barbata* sample. With a material-liquid ratio of 1:25 (g/ml), the sample was extracted twice with hot water at 90°C for 2 h each time. The mixture was centrifuged at 3,500 rpm for 15 min, combined with the supernatant, and concentrated by rotary evaporation at 55°C to 1/4 of the original volume. While stirring, 4 volumes of absolute ethanol were slowly added to the concentrated solution, and the solution was cooled to 4°C. The precipitate was collected, diluted with water, and deproteinized with Sevage reagent (the chloroform: n-butanol volume ratio was 4:1) (volume ratio 1:5) (Chen and Xue, 2019; Yao et al., 2020). The solution was decolorized with macroporous resin D101 and filtered and concentrated under reduced pressure at 55°C to 1/4 of its original volume. SBP was prepared by vacuum drying at 50°C and stored in a desiccator until use.

### SBP Purification

The crude polysaccharide was dissolved with distilled water to a concentration of 20 mg/ml and then injected into a DEAE-52 anionic cellulose chromatography column (2.5 cm × 100 cm). A gradient elution was performed with water and 0.1, 0.2, 0.3, 0.5, and 1 M NaCl solutions. The flow rate was adjusted to 1 ml/min, the eluent was collected at 10 ml/tube with an automatic collector, and the absorbance at 490 nm was measured with the phenol sulfuric acid method (Dubois et al., 1951). The elution fractions containing the same component were collected, concentrated to 1/8 of the original volume at 55°C; dialyzed with a dialysis bag with a molecular weight of 3,500 Da for 48 h, prefrozen at −40°C for 24 h, and then freeze-dried into powder (Zhang C. et al., 2019). Samples were placed on a SephadexG-100 dextran gel column and washed with ultrapure water, and the eluent was collected in a 5 ml tube. The polysaccharide content of each tube was measured by the phenol-sulfuric acid method, and elution curves were generated. The main peak components were concentrated, frozen and dried.

### Polysaccharide and Protein Content

First, 0, 0.4, 0.6, 0.8, 1.0, 1.2, 1.4, 1.6, and 1.8 ml of the glucose standard with a concentration of 0.1 mg/ml were placed in nine test tubes with stoppers, and distilled water was added to bring the volume up to 2 ml. Then, 1 ml of 5% phenol was added, and 5 ml of concentrated sulfuric acid was quickly added, followed by through mixing. The tubes were placed at 40°C for 30 min. After cooling to room temperature, the absorbance value at 490 nm was measured with an ultraviolet spectrophotometer; the glucose standard concentration (μg/ml) was used as the abscissa and the absorbance value A was used as the ordinate to draw the glucose standard curve. Then, 1 mg of SBP was weighed out and fully dissolved in distilled water. According to the phenol-sulfuric acid method (Dubois et al., 1951), the absorbance value at 490 nm was measured and substituted into the standard curve to calculate the polysaccharide concentration. Then, according to the BCA kit instructions, the BCA working solution was prepared, and 10 μl of the BSA standard was diluted to 100 μl (0.5 mg/ml) with phosphate-buffered saline (PBS). The standard (0, 2, 4, 6, 8, 12, 16, and 20 μl) was added to 96-well plates, and PBS was added to bring the volumes in the wells to 20 μl. A 20 μl sample diluted with PBS and 200 μl/well BCA working solution were added to the plates, which were then incubated at 37°C for 30 min. The absorbance value at 562 nm was measured by an enzyme labelling instrument (Multiskan MK3, Thermo Fisher, United States), and the protein concentration was calculated according to the standard curve.

### Monosaccharide Composition

Sixteen kinds of monosaccharide standards were added to test tubes at concentrations of 0.01, 0.1, 0.5, 1, 5, 10, and 20 mg/L. Ten milligrams of the sample was accurately weighed into an ampoule, and 10 ml of 3 M TFA was added to hydrolyse the substrate at 120°C for 3 h. The acid hydrolysis solution was aspirated, transferred into the tube, and dried with nitrogen. Next, 5 ml of water was added, and the mixture was vortexed, and diluted to 100 μl. Then, 900 μl of deionized water was added, and the mixture was centrifuged at 12,000 rpm for 5 min. The supernatant was removed, and a ICS5000 system (Thermo Fisher, Unites States) was used for ion chromatography (IC) analysis (Hui et al., 2019).

### Molecular Weight Determination

High-performance liquid chromatography (HPLC) is widely used for the determination of molecular weight due to its high precision and efficiency (Li et al., 2020). Samples and standards were accurately weighed (the molecular weights were 5,000, 11,600, 2,800, 48600, 80900, 148,000, 273,000, 409,800, 667,800, and 3693000 Da), and 5 mg/ml solutions were prepared and centrifuged at 12,000 rpm for 10 min. The supernatant was filtered through 0.22 μm microporous filters, and the sample was transferred to a 1.8 ml injection vial. The chromatographic conditions for HPLC (LC-10A, Shimadzu, Japan) were as follows: chromatographic column: BRT105-104-102 tandem gel column (8 × 300 mm); mobile phase: 0.05 M NaCl solution; flow rate: 0.6 ml/min, column temperature: 40°C; injection volume: 20 μl; detector: differential refractive index detector RID-1OA. Taking the logarithm of the molecular weight of the standard (log(Mw)) as the ordinate and the peak time (min) as the abscissa, regression fitting was performed on the curve to obtain the standard curve of the molecular weight distribution. The above chromatographic separation conditions were used to obtain a chromatogram of the sample, record the retention time of a single symmetrical peak, and calculate the molecular weight.

### FT-IR Analysis

The samples were analysed in pressed KBr pellets by Fourier transform infrared (FT-IR) spectroscopy (Chylińska et al., 2016). Five milligrams of polysaccharide was placed in a mortar with dried KBr, mixed and ground fully, pressed into a thin sheet, and scanned with a Thermo Scientific Nicolet iS5 FT-IR spectrophotometer (Thermo Nicolet Co., Madison, WI, United States) at 500–4,000 cm^−1^.

### Anti-Hepatoma Activity of SBP-2A *In Vitro*

#### MTT Assay

The MTT assay was used to screen the SBP component with the highest antitumor activity. HepG2 cells were inoculated in a 96 well microplate, warmed in an incubator to 37°C under 5% CO_2_ to adhere to the wall, and cultured with different concentrations (50–3,200 μg/ml) of SBP, SBP-1A, and SBP-2A for 48 h. Then, 20 μl/well of MTT solution (5 mg/ml) was added, and the cells were cultured for 4 h. Then, the liquid was discarded, and 150 μl/well DMSO was added. The plate was slowly oscillated for 10 min, and the absorbance (OD) value was measured at 490 nm with a SpectraMax M3 microplate reader (Molecular Devices, USA). A_0_ represents the OD value of the black control group (10% FBS-MEM complete medium), A_1_ represents the OD value of the experimental group, and A_2_ represents the OD value of the negative control group.

The viability of HepG2 cells = (A_1_−A_0_)/(A_2_−A_0_)×100%

The IC_50_ values of the three polysaccharides at different concentrations were calculated by GraphPad Prism software. According to the IC_50_ value, the low, the medium, and the high doses of SBP-2A were used to study the anti-hepatoma effect.

#### Colony Formation Assay and Cell Morphology Observation

HepG2 cells were diluted and seeded at 4,000 cells/well in 35 mm dishes. After 10 h, the cells were treated with different concentrations of SBP-2A (0, 200 μg/ml, 400 μg/ml, 800 μg/ml) for 48 h and then cultured under standard conditions for 10 days. The MEM medium was replaced every 3 days. Finally, colonies were stained with 0.1% crystal violet, and colonies with more than 50 cells were counted. The colony formation assay was used to determine the colony formation ability of HepG2 cells treated with different concentrations of SBP-2A.

Colony formation rate (%) = number of cell colonies/number of inoculated cells.

HepG2 cells in the logarithmic growth stage were cocultured with SBP-2A for 48 h. The morphological changes in HepG2 cells were directly observed with a DMI 6000B Leica microsystem. Then, the culture medium was aspirated, 0.5 ml of fixing solution was added, and the cells were fixed for 20 min. Then, the cells were washed with PBS, cultured with Hoechst 33258 staining solution for 10 min, and rinsed with PBS. After blocking with an fluorescence quencher, the morphological changes in HepG2 cell apoptosis induced by SBP-2A were observed under a DMI 6000B Leica microsystem.

#### 5-Ethynyl-2′–Deoxyuridine Assay

HepG2 cells were inoculated into 12-well plates containing sterile cell climbing tablets and then cultured in an incubator with SBP-2A for 48 h. According to the instructions provided with the BeyoClick™ EdU Cell Proliferation Kit with Alexa Fluor 594 (Beyotime, C0078S), an equal volume of EdU solution (20 μM) was added, and the cells were incubated for 2 h, followed by fixing with 4% paraformaldehyde. Then, 0.3% Triton X-100 was added at 200 μl/well for cell permeabilization (Solarbio, T8200), and the cells were placed in 1 × Apollo 567 reaction solution at room temperature for 30 min. Then, 2-(4-amidinophenyl)-6-indolecarbamidine dihydrochloride (DAPI, Beyotime, C1005) was used to counterstain the cells for 10 min. Finally, the images were captured by the DMI 6000B Leica microsystem in a live cell imager (Wetzlar, Germany), and the percentage of EdU-positive cells in each group and each well was randomly counted in five fields of view.

#### Cell Cycle Detection

The cell cycle distribution of the HepG2 cell line treated with SBP-2A was evaluated by flow cytometry. The cells were seeded at a density of 5 × 10^5^ cells/well and inoculated in 35 mm cell culture dishes. After adding adherent, the cells were starved with MEM culture medium containing 1% FBS for 12 h. The cells were treated with SBP-2A solutions for 48 h. With a sample collection rate of 1 × 10^6^ cells/piece, cell precipitates were obtained by centrifugation, and resuspended by adding 300 μl of precooled PBS, slowly adding 700 μl of precooled absolute ethanol with mixing, and then fixing the cells at −4°C overnight. The mixture was warmed in 37°C water for 30 min, 400 μl of PI staining solution was added to mixture, and the mixture was incubated at 4°C in the dark for 30 min. Finally, the cell cycle of the sample was detected by a Cytomics FC500 flow cytometry instrument with a CXP system (Beckman, United States), and the data were analysed by ModFit LT 5.0 software.

#### Real-Time Quantitative Polymerase Chain Reaction Assay

HepG2 cells were treated with different concentrations of SBP-2A for 48 h; the cells were lysed with TRIzol reagent (Solarbio, China) and total RNA was extracted. According to the manufacturer’s protocol, the total RNA was reverse transcribed into cDNA by using an MMLV Reverse Transcriptase RNaseH-kit (Promega, United States) with 500 ng total RNA as a template. Real-time quantitative PCR was completed by an MX3000P real-time PCR Instrument (Stratagene, United States) and SYBR Real-time PCR Universal Reagent (Aibosi, Shanghai). The mRNA expression levels of P53 and CyclinD1 were standardized to glyceraldehyde 3-phosphate dehydrogenase (GAPDH) level. The specific primer sequences were designed by Shanghai Aibosi Biotechnology Co., Ltd., and are shown in the table below.

**Table:** 

Gene Name	Primer Name	Primer sequence (5’>3′)
GAPDH	F primer	CAT​GAG​AAG​TAT​GAC​AAC​AGC​CT
	R primer	AGT​CCT​TCC​ACG​ATA​CCA​AAG​T
	Product	113 bp
P53	F primer	CTA​TGA​GCC​GCC​TGA​GGT​T
	R primer	AGC​TGT​TCC​GTC​CCA​GTA​GAT
	Product	152 bp
CCND1	F primer	AAC​AAA​CAG​ATC​ATC​CGC​AAA​CAC
	R primer	GTT​GGG​GCT​CCT​CAG​GTT​CAG
	Product	144 bp

#### Western Blot Assay

The HepG2 cells were cocultured with SBP-2A solution for 48 h. Then, the HepG2 cells and tumour tissue were lysed completely in a powerful RIPA (Solarbio, R0010, China) lysis buffer supplemented with protease and phosphatase inhibitors and centrifuged at 14000 × *g* for 5 min at 4°C. The total protein concentration was determined according to the instructions of a BCA protein quantitative kit (Beyotime Biotechnology, P0010, China). The total protein was separated by sodium dodecyl sulfate–polyacrylamide gel electrophoresis (SDS–PAGE) and transferred to 0.45 µm methanol-activated polyvinylidene fluoride (PVDF) membranes (Beyotime Biotechnology, Shanghai, China), followed by blocking with QuickBlock blocking buffer (P0252, Beyotime) at room temperature for 15 min. Next, diluted primary antibodies against *β*-actin (1:1,000, 42 kDa, Beyotime, AF5003), P53 (1:1,000, 53 kDa, Wanleibio, WL01919), CyclinD1 (1:1,000; 35 kDa, Wanleibio, WL01435a), CDK4 (1:500, 34 kDa, Wanleibio, WL02274), Bax (1:10000, 23 kDa, Proteintech, 60267-1-lg) and Bcl-2 (1:500, 26 kDa, Beyotime, AF6285) were added and the membranes were incubated overnight at 4°C. The membranes were washed with TBST solution and then incubated with a diluted horseradish peroxidase-bound secondary antibody (1:5,000, Beyotime, A0208) for 1 h at 25°C. After washing with TBST solution, the bands were detected by Super-GL ECL hypersensitive luminescent solution and photographed with a hypersensitive multifunctional imager. Taking the grey value of the *β*-actin protein band as a reference, the protein bands of P53, CyclinD1, CDK4, Bax, and Bcl-2 were quantitatively analysed by ImageJ software.

### Antitumour Activity of SBP-2A *In Vivo*

C57BL/6 mice were purchased from Liaoning Changsheng Biotechnology Co., Ltd. (Liaoning, China; Laboratory animal welfare and ethical review number: 20220314-1). The study was approved by the Ethics Committee of Mudanjiang Medical University (Mudanjiang, China). C57BL/6 male mice were raised in room without specific pathogen-free (SPF) conditions (Mudanjiang Medical University, Mudanjiang, China), and weighed 20 ± 2 g. H22 cells were cultured with RMPI 1640 complete medium, cells in the logarithmic growth phase were adjusted to 1.5 × 10^7^ cells/mL, and washed with PBS 3 times. Then, the cells were suspended in 200 μl of PBS and injected subcutaneous into the right armpit of 30 C57BL/6 mice. The mice were randomly divided into 5 groups (*n* = 6 per group), included the model group, SBP-2A group (25, 50, and 100 mg/kg), and *Astragalus* polysaccharide (APS) group. On the second day after modelling, the model group was injected with saline and the treatment group was injected with SBP-2A via the peritoneal cavity, once a day for 12 consecutive days. On day 13, the mice were sacrificed, and the tumors were excised and weighed, the tumor volume was measured. The tumour inhibition rate of each group was calculated.

The tumor inhibition rate (%) = [1−(tumor weigh of the SBP-2A group/tumor weigh of the model group)] ×100%

### Statistical Analyses

All data are expressed as the mean ± standard deviation (SD). All experiments were repeated three times. One-way ANOVA and multiple comparisons were used, and all statistical analyses were performed by using GraphPad Prism 7.0 software and plotted with Origin 2021. *p* < 0.05 was taken to indicate a statistically significant difference.

## Results

### Single-Factor Design of SBP Extraction Conditions

**FIGURE 1:**
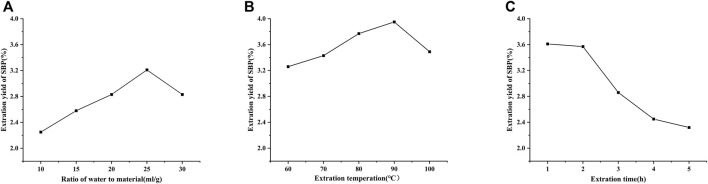
Effects of different material-liquid ratios **(A)**, extraction time **(B)**, and extraction temperatures **(C)** on the SBP extraction rate.

Figure 1 shows the effects of different extraction parameters on the efficiency of the extraction of SBP. Figure 1A shows the effects of material-liquid ratios of 1:10, 1:15, 1:20, 1:25, and 1:30 on the extraction of SBP. The rates of polysaccharide extraction increased with increasing material-liquid ratio. When the material-liquid ratio was more than 1:25, the extraction rate increased rapidly, possibly because the increase in the material-liquid ratio allowed the polysaccharides to dissolve more fully (Wu et al., 2020; Bargougui et al., 2019). Therefore, 1:25 was chosen as the center of the response surface experiment. As shown in Figure 1B, the effects of 60, 70, 80, 90 and 100°C on the extraction of SBP were investigated. When the extraction temperature was 90°C, the polysaccharide extraction rate was highest. When the extraction temperature was increased from 60 to 90°C, the rate of polysaccharide extraction increased. As the temperature increased further, the extraction rate showed a decreasing trend. Therefore, the extraction temperature of 90°C was chosen as the central point of the response surface experiment. As shown in Figure 1C, when the extraction time was 1 h or 2 h, the rate of SBP extraction was higher, and extending the extraction time increased the polysaccharides extraction rate (Mkadmini Hammi et al., 2016); the rate of SBP extraction decreased as the time increased from 2 to 4 h, and it is possible that the structures of the polysaccharides were changed during the long-term extraction process (Ma et al., 2016). Therefore, 2 h was chosen as the centre of the response surface experiment.

### Analysis of Response Surface Optimization Results

#### Regression Model and Analysis of Variance

A response surface optimization experiment with three factors and three levels was designed based on the results of the single-factor experiment. The polysaccharide extraction rate was the response value, and the material-liquid ratio (A), extraction temperature (B), and extraction time (C) were used as influencing factors. The Design Expert 11 software was used to process the data. As shown in Table 1, a binary multiple equation relating the extraction rate of SBP (y) and the three factors was obtained by data analysis:

**TABLE 1:** Response surface experimental design and results.

Rang	A	B	C	Y
	Ratio of Water to Material (g/ml)	Extraction Time (h)	Extraction Temperature (°C)	Extraction Rate of SBP (%)
1	30	−1	0	2.64
2	25	1	1	8.84
3	25	1	−1	3.77
4	20	−1	0	2.42
5	25	0	0	3.97
6	30	0	−1	2.83
7	20	0	1	2.88
8	25	−1	1	3.81
9	30	1	0	2.74
10	25	0	0	3.9
11	25	−1	−1	3.27
12	25	0	0	3.95
13	20	1	0	2.79
14	25	0	0	3.91
15	20	0	−1	2.93
16	25	0	0	3.82
17	30	0	1	3.17

Y = 3.91 + 0.045A + 0.125B + 0.113C−0.068AB + 0.098AC−0.118BC−0.991A2−0.271B2 + 0.034C2

Y is the SBP extraction efficiency; A is the ratio of material to liquid; B is the extraction time; and C is the extraction temperature.

Significance tests were conducted for all models and regression model coefficients, and the results are shown in Table 2. The F value of the model was 143.86, and the *p* value was <0.0001, which indicates that the regression model had very high significant; the model mismatch term *p* value was 0.3838 (*p* > 0.05), so it was not significant at the level of *α* = 0.05; this indicates that the model fitting was effective, and the experimental error was small. The correlation coefficient *r* was 0.9946, indicating that the simulated value of the model was consistent with the actual predicted value, and the prediction of the model was reasonable; the coefficient of variation (CV) was only 1.85%, so the model had good repeatability and high accuracy. From the F values of the three influencing factors (A, B, and C), it can be concluded that the effects of the influencing factors on the extraction rate of SBP decreased in the following order: extraction time > extraction temperature > material-liquid ratio.

**TABLE 2:** Regression Model and analysis of variance.

Source	Sum of squares	DF	Mean square	*F*-value	*p*-value
Model	4.94	9	0.55	143.86	<0.0001
A-Ratio of water to material	0.016	1	0.016	4.25	0.0783
B-Extraction time	0.12	1	0.13	32.77	0.0007
C-Extraction temperature	0.10	1	0.10	26.54	0.0013
AB	0.018	1	0.018	4.78	0.0651
AC	0.038	1	0.038	9.97	0.0160
BC	0.055	1	0.055	14.48	0.0067
A^2^	4.14	1	4.14	1,084.65	<0.0001
B^2^	0.31	1	0.31	81.22	<0.0001
C^2^	0.005	1	0.005	1.26	0.2991
Residual	0.028	7	0.004		
Lack of Fit	0.013	3	0.004	1.32	0.3838
Pure Error	0.013	4	0.003		
Cor Total	4.97	16			

#### Response Surface Analysis and Model Optimization

**FIGURE 2:**
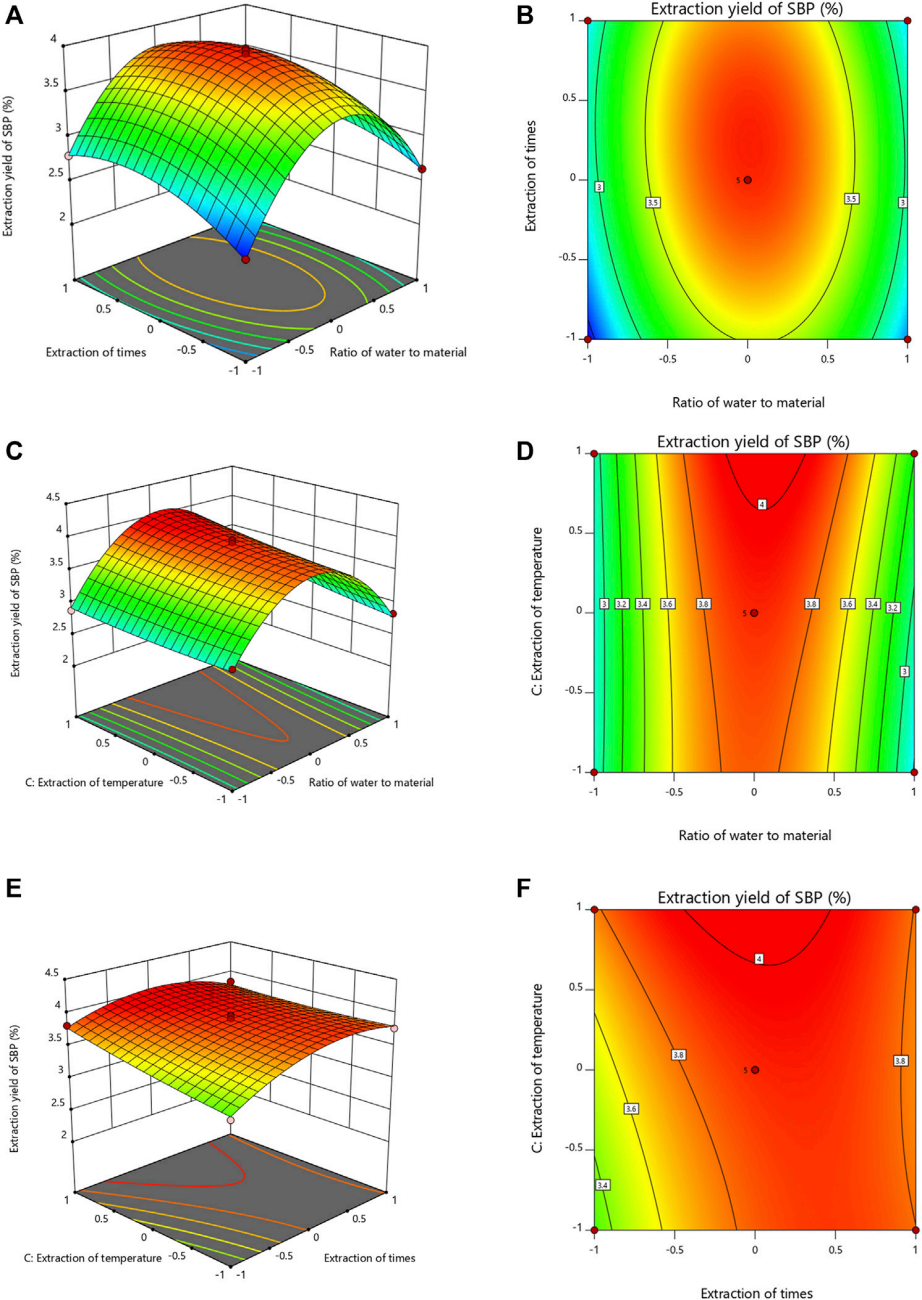
Response surface and contour map of the three-factor interaction in the SBP extraction rate. **(A–F)** showing the effects of extraction time, ratio of water to material and extraction temperature on the extraction yields of SBP.

The response surface diagram and the contour map obtained by the multiple quadratic regression models were used to evaluate the pairwise interactions of experimental factors and their impact on the extraction of SBP, which determined the optimal level range for each factor. The steeper the slope of the response surface is, the higher the response sensitivity. The shape of the contour line reflects the strength of the interaction. The contour lines for the material-liquid ratio and the extraction time tended to be oval, indicating that the interactions were significant; the interactions with other factors were not significant (Figure 2). The optimal extraction parameters for SBP were a material-liquid ratio of 1:25.36, an extraction time of 120.3 min, and an extraction temperature of 100°C.

### SBP Purification

**FIGURE 3:**
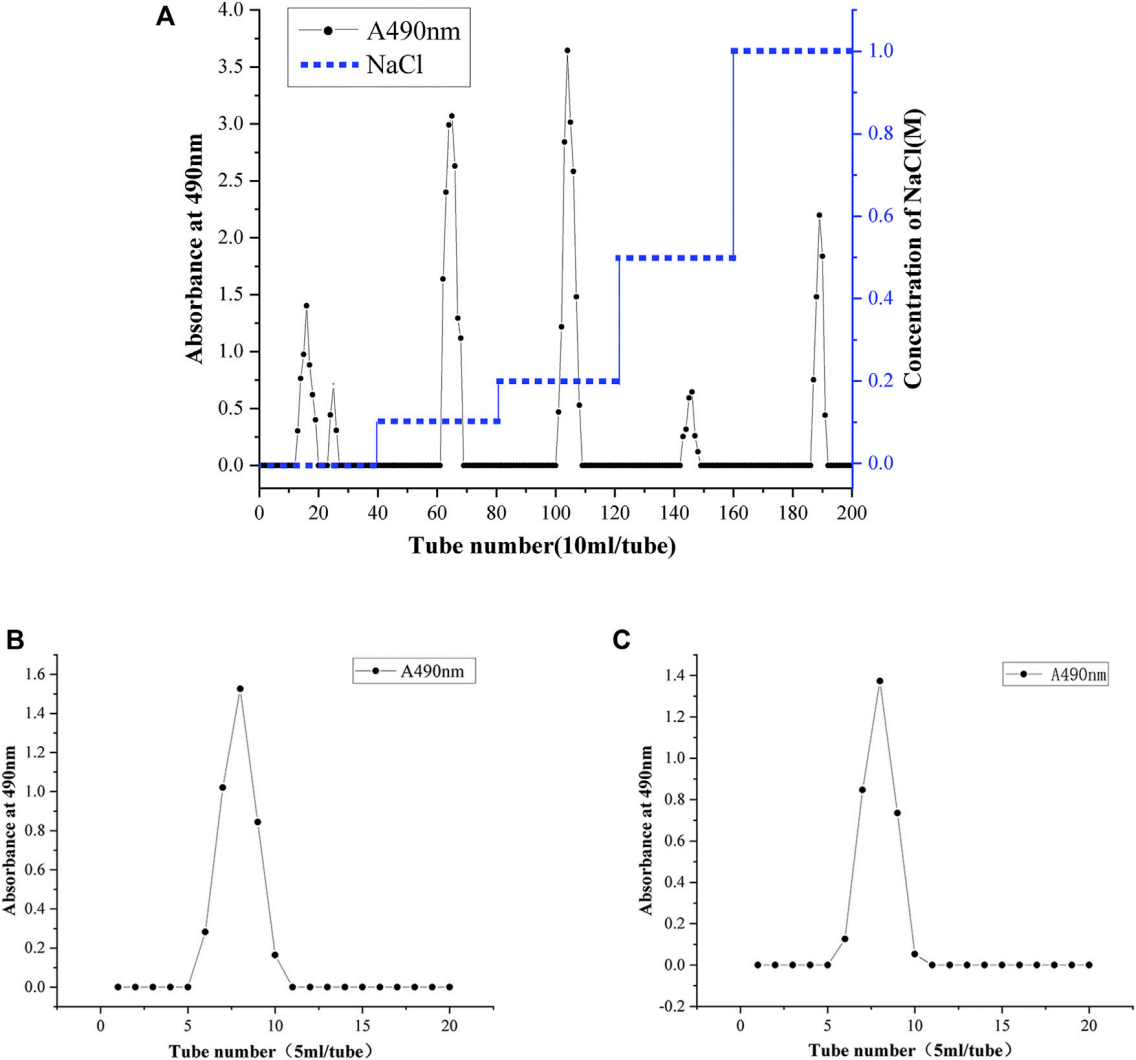
Gradient elution profile of SBP on a DEAE-52 anion cellulose chromatography column with NaCl solutions (0, 0.1, 0.2, 0.5, and 1 M) **(A)**. Elution profiles of SBP-1A **(B)** and SBP-2A **(C)** on a Sephadex G-100 gel chromatography column with deionized water.

Ion chromatography and gel column chromatography are usually used for the separation and purification of polysaccharides. The polysaccharide fractions with single peaks obtained by eluting with 0.1 and 0.2 M NaCl solutions were the largest (Figure 3A). Therefore, the eluates with these two elution peaks were collected and named SBP-1 and SBP-2. The two components SBP-1 and SBP-2 were purified by a SephadexG-100 gel column. As shown in Figures 3B,C, the two components showed a single and symmetrical elution peak respectively, demonstrating that both polysaccharides were relatively homogeneous; the samples with the single elution peak were collected and lyophilized to obtain white flocculent powders, which were named SBP-1A and SBP-2A.

### Chemical Composition Analysis

#### Determination of Polysaccharide Content and Protein Content

Analysis showed that the polysaccharide content of SBP-1A was 93.2% and that of SBP-2A was 95.5%. A standard curve was obtained by using BSA protein standards, and the equation of the curve was *y* = 1.1702x + 0.1401, *R* ^2^ = 0.9902. The protein contents of the polysaccharide samples were determined: the protein content of SBP-1A was 2.87% and that of SBP-2A was 0.87% (Table 3).

**TABLE 3:** Preliminary characterization of SBP-1A and SBP-2A.

Sample	Total Sugar (%)	Uronic Acids (%)	Mw (Da)	Monosaccharide Composition (molar Ratio)						
				Rha	Fuc	Ara	Xyl	Man	Glc	Gal
SBP-1A	93.2	0.7	1.15 × 105	0.6	0.6	30.6	8.0	1.4	16.1	38.4
SBP-2A	95.5	1.2	1.4 × 105	0.8	0.6	36.3	3.6	0.7	9.2	42.7

#### Monosaccharide Composition

**FIGURE 4:**
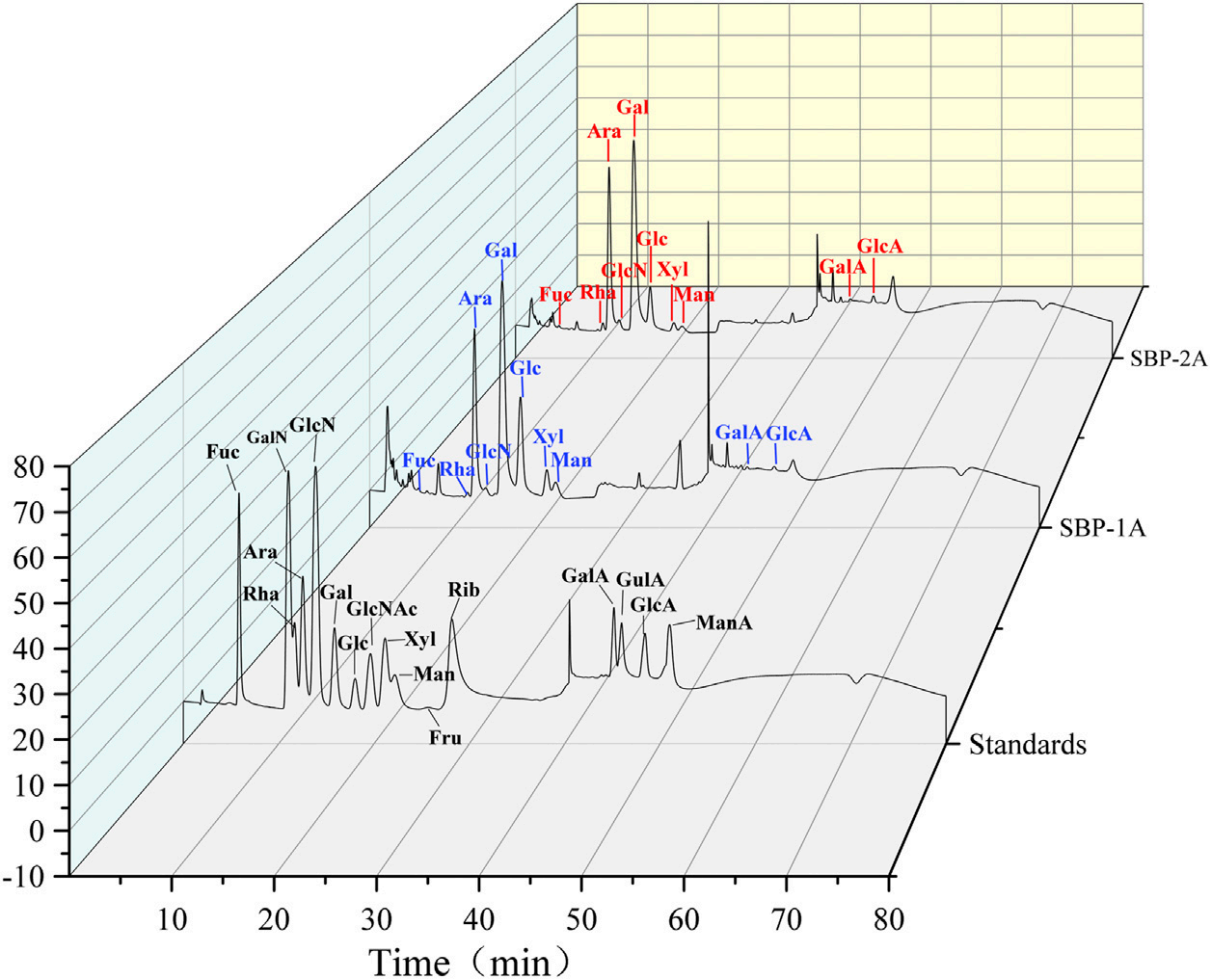
IC chromatogram of mixed monosaccharide standards SBP-1A and SBP-2A.

The monosaccharide compositions of SBP-1A and SBP-2A were analysed by IC. The peak sequences and retention time of the monosaccharide compositions were compared with those in chromatograms for the monosaccharide standard samples (Figure 4). For the mixed standard, the peak at 2.0 min represented sodium hydroxide, and the peak at 40 min represented sodium acetate. Table 3 shows the molar ratios of the monosaccharide samples. SBP-1A consisted mainly of arabinose (30.6%) and galactose (38.4%), and the uronic acid content was 0.7%; SBP-2A consisted mainly of arabinose (36.3%) and galactose (42.7%), and the uronic acid content was 1.2%. The monosaccharide constituents of the two components were fucose, galactosamine hydrochloride, rhamnose, arabinose, glucosamine hydrochloride, galactose, glucose, xylose, and mannose, but the molar ratios were different: the molar ratio for SBP-1A was 0.6:0.3:0.6:30.6:3.3:38.4:16.1:8:1.4, and the molar ratio for SBP-2A was 0.6:0.5:0.8:36.3:4.4:42.7:9.2:3.6:0.7.

#### Molecular Weight Determination

**FIGURE 5:**
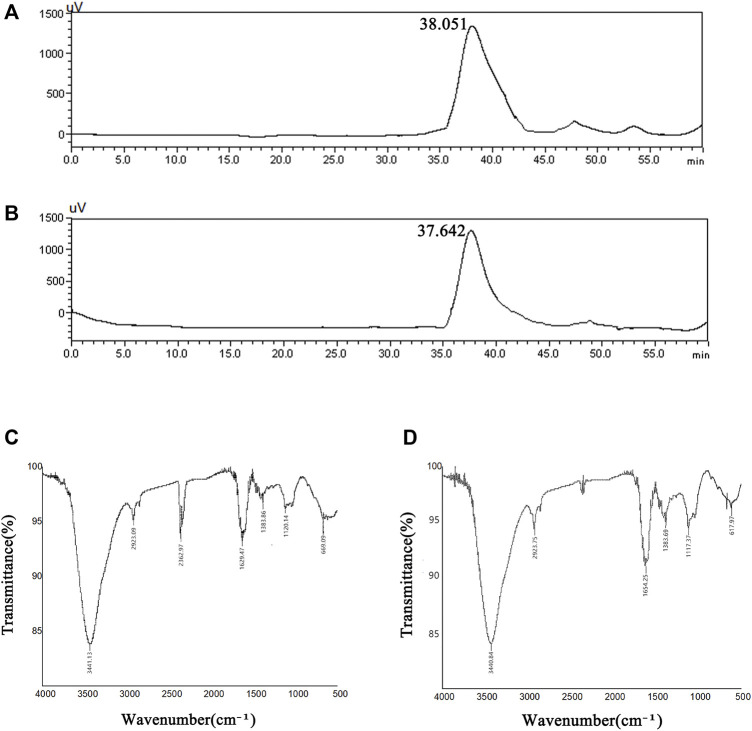
HPLC profiles of SBP-1A **(A)** and SBP-2A **(B)**. FT-IR spectra of SBP-1A **(C)** and SBP-2A **(D)**.

The molecular weight distributions of SBP-1A and SBP-2A were determined by HPLC, and the following standard curve equation was obtained: lg Mw = −0.2078x + 12.968, *R* ^2^ = 0.993. Figures 5A,B shows a chromatogram with two polysaccharide components. Both SBP-1A and SBP-2A showed symmetrical single peaks, indicating that they were homogeneous acidic polysaccharides with high purity. The molecular weight of SBP-1A was 1.15 × 105 Da (retention time: 38.051 min), and the molecular weight of SBP-2A was 1.4 × 105 Da (retention time: 37.642 min).

#### FT-IR Infrared Spectroscopy

As shown in Figures 5C,D, the FT-IR analysis showed that SBP-1A and SBP-2A had characteristic absorption peaks for polysaccharides near 3,400 cm^−1^, and the strong absorption peak was the result of O-H stretching vibrations. The peak near 2,900 cm^−1^ was assigned to C-H stretching vibrations. There was a stretching vibration for C=O at 1,634 cm^−1^, which may have been due to a carboxyl or acetyl group, indicating the presence of uronic acid. The absorption peak near 1,380 cm^−1^ was caused by the variable angle vibration of C-H, indicating that the polysaccharide had the *β*-characteristic absorption peak of dextran. A peak appeared near 1,120 cm^−1^, indicating that the sugar residue of the polysaccharide was mainly pyranose.

### Anti-Hepatoma Activity of SBP-2A *In Vitro*

#### MTT Assay

**FIGURE 6:**
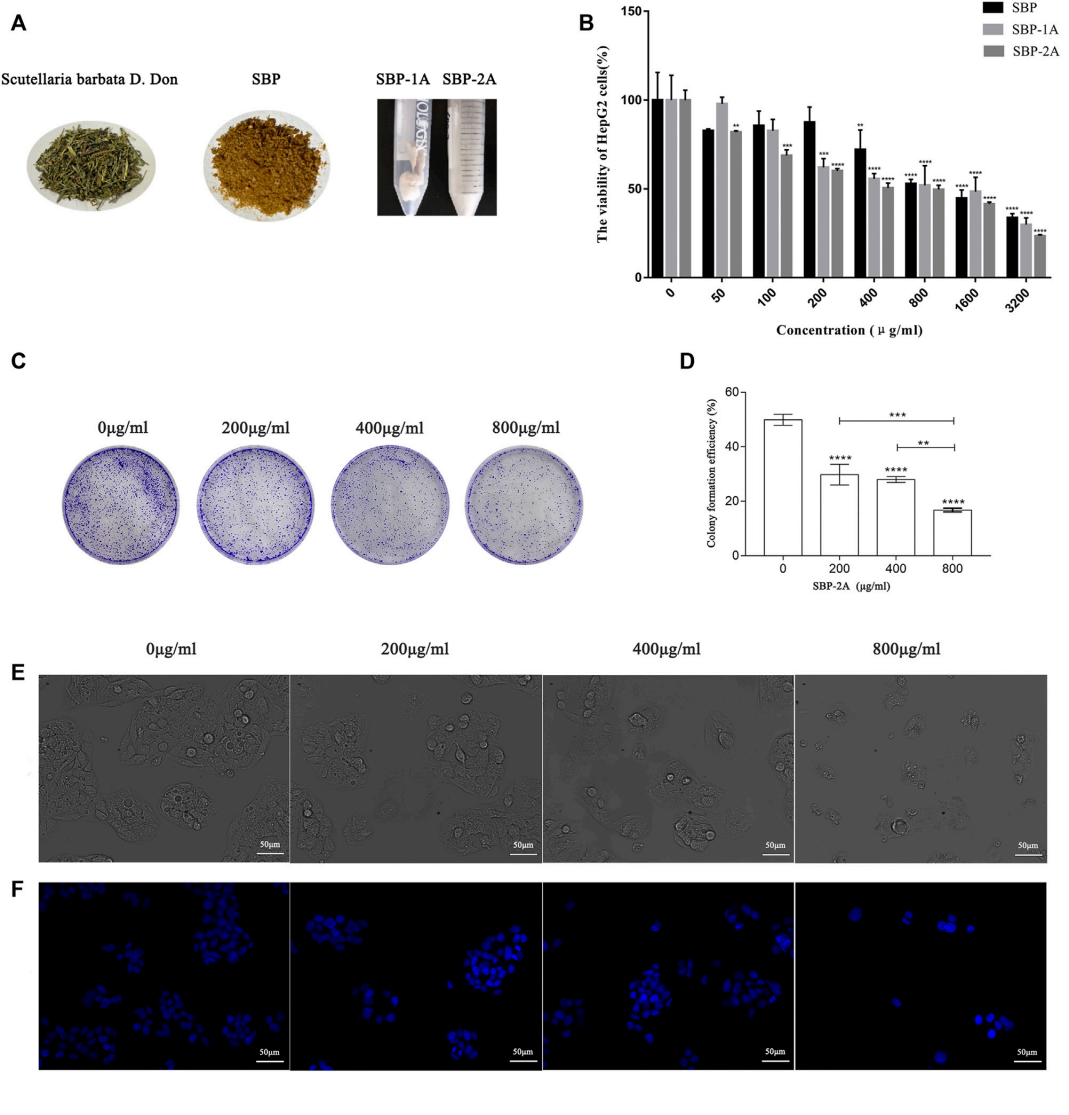
**(A)** *Scutellaria barbata* D. Don, SBP, SBP-1A, and SBP-2A. The viability of HepG2 cells treated with SBP, SBP-1A and SBP-2A (0–3,200 μg/ml) for 48 h was measured by MTT assay **(B)**. Colony formation assays **(C,D)**, in contrast to the non-SBP-2A-treated group, ^**^ *p* < 0.01, ^***^ *p* < 0.001, ^****^ *p* < 0.0001. The morphological characteristics **(E)** and Hoechst 33258 staining **(F)** of HepG2 cells treated with different concentrations of SBP-2A for 48 h.

To identify the *in vitro* antitumour activities of isolated and purified SBP, the cell viability of HepG2 cells treated with different concentrations of *S. barbata* polysaccharides (SBP, SBP-1A, and SBP-2A) for 48 h was evaluated by the MTT method. Figure 6B shows that the inhibition of HepG2 cells growth, increased with increasing SBP concentration. The average inhibition rates for SBP, SBP-1A and SBP-2A increased from 17.29 to 66.35%, 2.13–70.19% and 18.02–76.53%, respectively. Compared with SBP, SBP-1A and SBP-2A significantly inhibited the growth of HepG2 cells in the dose range of 50–400 μg/ml, with IC_50_ values of 891.7 μg/ml and 548.3 μg/ml after 48 h. We found that when the concentration was 400 μg/ml, the average inhibition rate of SBP-2A for HepG2 cells was 49.52%, which was significantly higher than that of SBP-1A. Therefore, SBP-2A at concentrations of 200, 400, and 800 μg/ml was chosen for subsequent anti-hepatoma activity experiments. Figures 6C,D shows that the numbers of cell colonies formed by HepG2 cells treated with different concentrations of SBP-2A were significantly reduced compared with that of the control group. When the concentration was 800 μg/ml, the number of colonies formed decreased most significantly, indicating that SBP-2A significantly inhibited hepatoma cells colony formation; this was consistent with the results of the MTT assay.

#### Morphological Changes in Apoptosis Induced by SBP-2A

As illustrated in Figure 6E, HepG2 cells with normal morphologies showed irregular spindle shapes, high numbers, full shapes and clustered adherent growth. Compared with the control group, HepG2 cells gradually lost their original normal cell morphology with increasing SBP-2A concentrations, which showed that the number of cells decreased significantly, the cells became flat and contracted into a lump, and the cell membranes were broken, which resulted in unclear boundaries between the cells and cell fragments. These results showed that SBP-2A significantly inhibited the proliferation of HepG2 cells *in vitro*. Figure 6F shows that HepG2 cells exhibited typical apoptotic characteristics compared with the control group, such as dense nuclear staining and an increased bright blue density after 48 h of treatment with SBP-2A. However, with increasing SBP-2A concentration, the number of bright blue spots first increased and then decreased, preliminarily indicating that SBP-2A induced apoptosis of HepG2 cells.

#### EdU Assay

**FIGURE 7:**
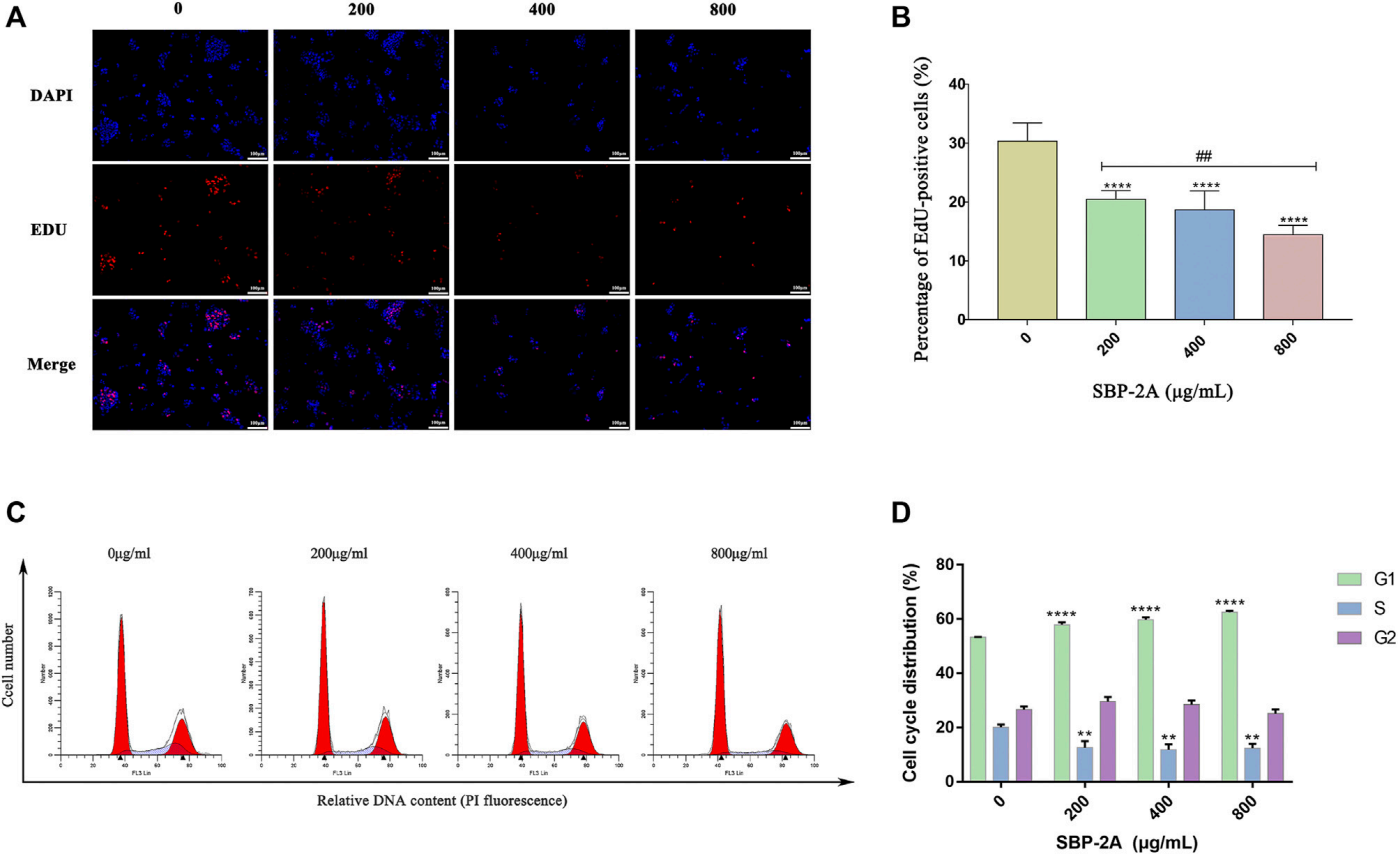
Edu staining **(A)**, and Edu-positive (+) cells were counted by ImageJ software **(B)**. Compared to the non-SBP-2A-treated group, ^****^ *p* < 0.0001. The number of apoptotic cells was determined by Annexin V-FITC/PI double staining **(C)**, and the DNA contents in the cell cycle was measured quantitatively by flow cytometry **(D)**.

An EdU assay was used to further evaluate the effect of SBP-2A on the proliferation of HepG2 cells. The nuclei of the proliferating cells were stained red by EdU and stained blue by DAPI (Figure 7A). Compared with the negative control group, the percentage of EdU-positive cells decreased significantly with increasing drug concentration (*p* < 0.05) (Figure 7B). This resultindicated that SBP-2A significantly inhibited the proliferation of HepG2 cells.

#### DNA Content Quantitation Assay

As displayed in Figure 7C, by analysing the percentage of DNA content in G0/G1, S and G2/M cells in the cell cycle, we found that compared with the untreated group, SBP-2A increased the percentage of cells in the G1 phase from 53.34 ± 0.06% to 62.51 ± 0.54% (*p* < 0.0001), and the percentage of cells in the S phase was decreased from 20 ± 1.1% to 12.34 ± 1.66% (*p* < 0.01), indicating a dose-dependent effect; the effect in cells in the G2/M phase was statistically insignificant (Figure 7D and Table 4). This observation was accompanied by an increase in the number of cells in the G0/G1 phase. HepG2 cells were blocked by SBP-2A in the G0/G1 phase of the cell cycle.

**TABLE 4:** Effect of SBP-2A on the cell cycle distribution of HepG2 cells for 48 h (*n* = 3).

Cell Cycle (%)	Concentration (μg/ml)			
	0	200	400	800
G1	53.34 ± 0.06	57.92 ± 0.94^****^	59.77 ± 0.77^****^	62.51 ± 0.54^****^
S	20 ± 1.1	12.55 ± 2.44^**^	11.82 ± 2.01^**^	12.34 ± 1.66^**^
G2\M	26.66 ± 1.14	29.53 ± 1.7	28.41 ± 1.55	25.15 ± 1.45

Note: HepG2 cells were treated with SBP-2A (200 μg/ml, 400 μg/ml, 800 μg/ml) for 48 h (Mean ± SD, ^**^ *p* < 0.01, ^****^ *p* < 0.0001 vs. non-SBP-2A-treated group).

#### qRT-PCR and Western Blot Analysis

**FIGURE 8:**
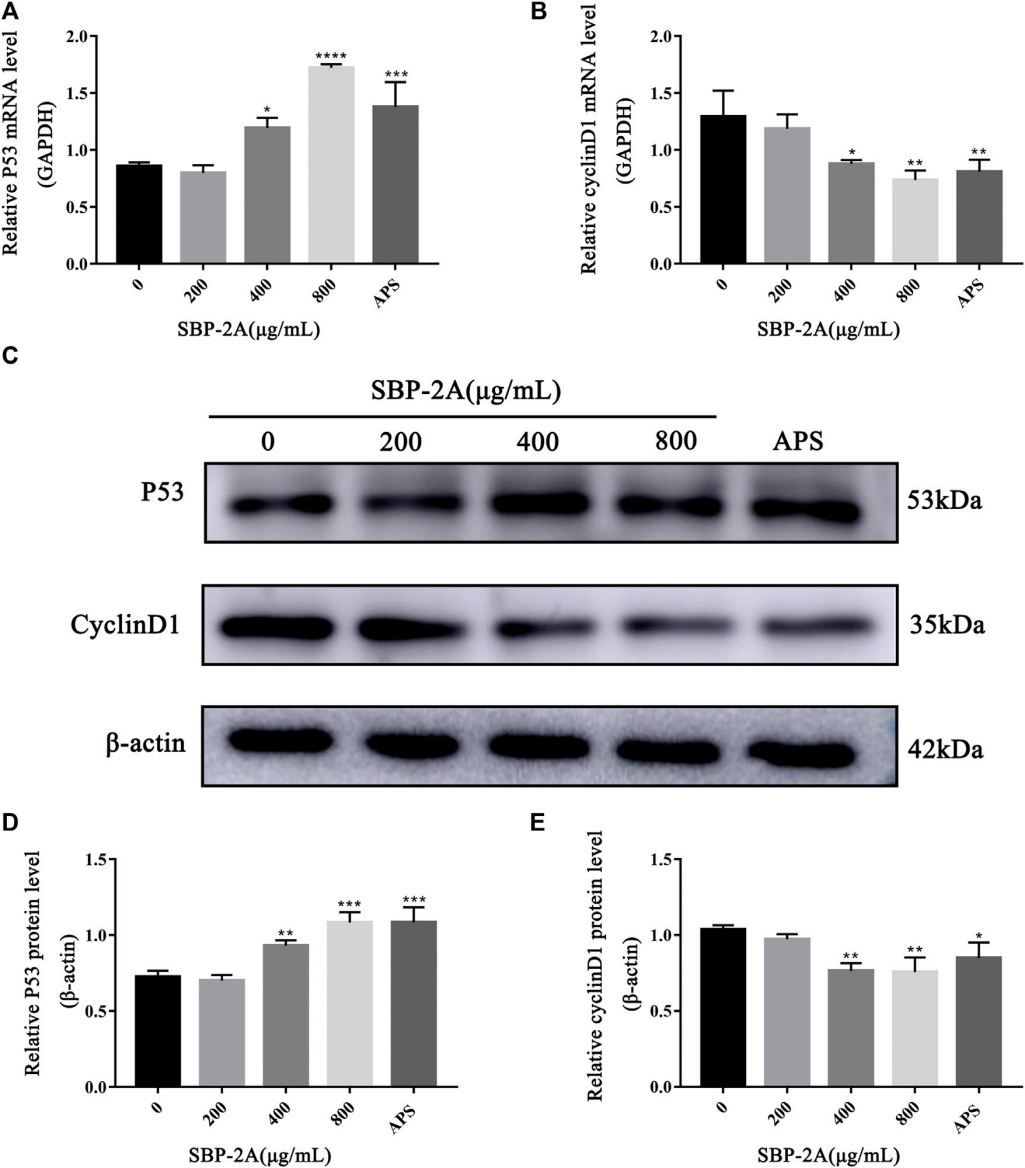
The effect of SBP-2A on the mRNA expression of P53 and CyclinD1 was determined by qRT–PCR **(A,B)**. Compared to the non-SBP-2A-treated group, ^*^ *p* < 0.05, ^**^ *p* < 0.01, ^***^ *p* < 0.001, ^****^ *p* < 0.0001. The protein expression of p53 and cyclin D1 **(C–E)**. Compared to the non-SBP-2A-treated group, ^*^ *p* < 0.05, ^**^ *p* < 0.01, and ^***^ *p* < 0.001.

**FIGURE 9:**
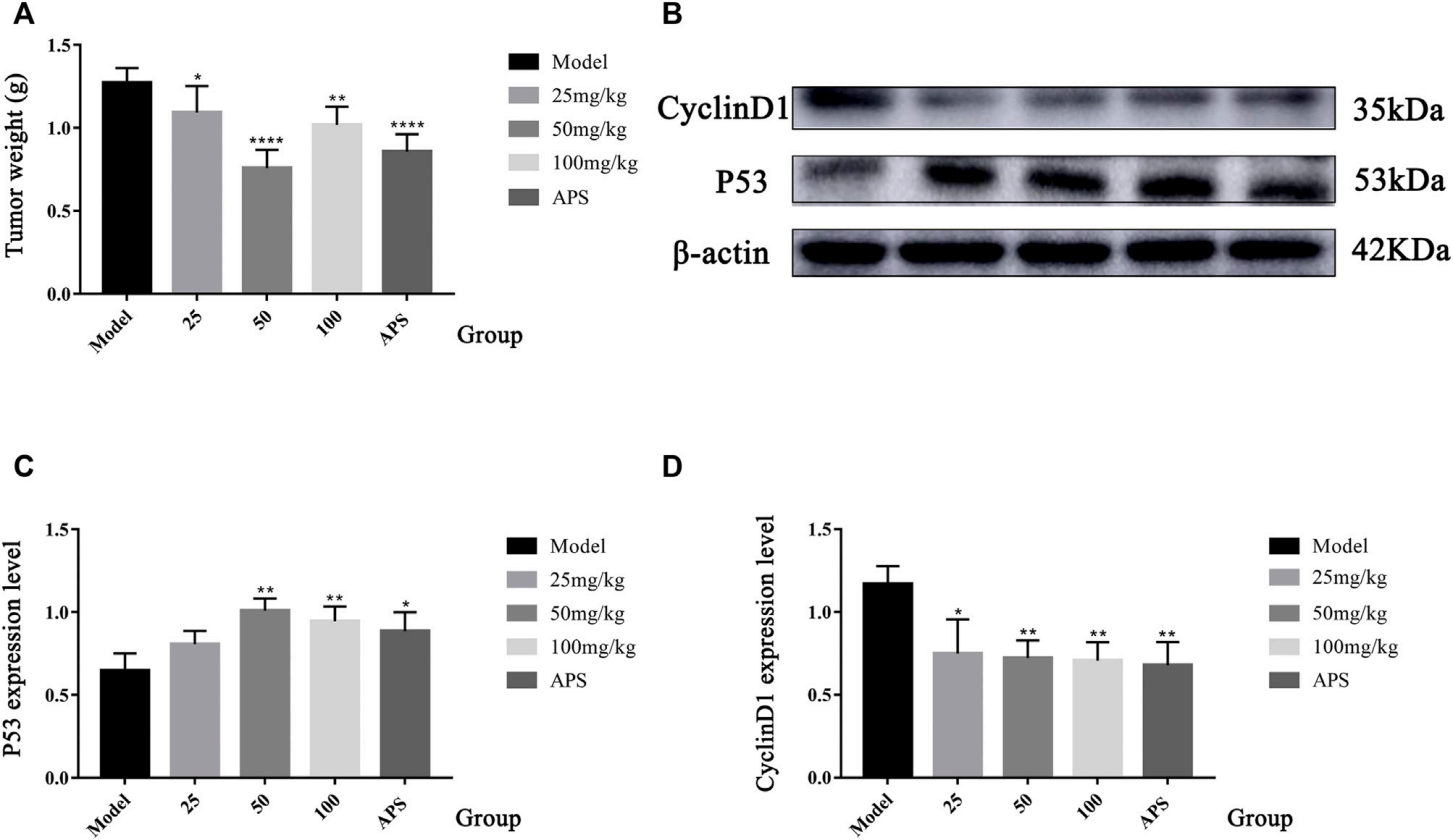
Tumour weight of H22 tumour-bearing mice **(A)**. Compared to the model group, ^*^ *p* < 0.05, ^**^ *p* < 0.01, and ^****^ *p* < 0.0001. The protein expression of P53 and CyclinD1 **(B–D)**. Contrast to the model group, ^*^ *p* < 0.05, and ^**^ *p* < 0.01.

**FIGURE 10:**
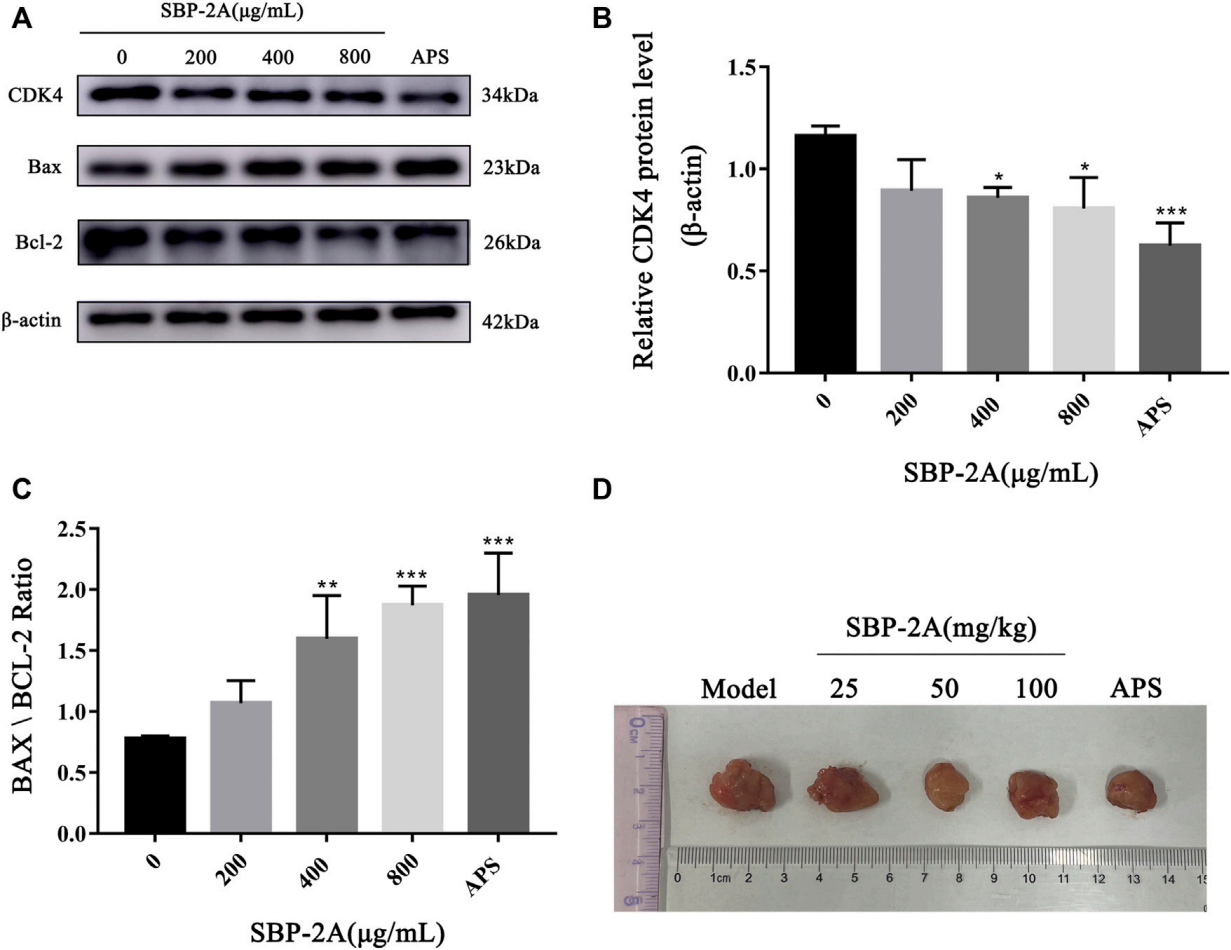
The protein expression of CDK4, Bax and Bcl-2 **(A–C)**. Contrast to the without SBP-2A group, ^*^ *p* < 0.05, ^**^ *p* < 0.01, ^***^ *p* < 0.001. H22 mice tumor **(D)**.

Cell cycle progression is regulated by cell cycle-related molecules. We found that HepG2 cells were blocked by SBP-2A in the G1 phase of cell synthesis. In addition, P53 and CyclinD1 are the main genes that regulate the G1 phase of the cell cycle. Therefore, after treating HepG2 cells with different concentrations of SBP-2A for 48 h, we examined the relative expression levels of cyclin D1 and P53 mRNA by qRT-PCR (Figures 8A,B). Compared with the group without SBP-2A, there was no significant difference between the 200 μg/ml group and the negative group, but 400 μg/ml and 800 μg/ml SBP-2A significantly downregulated the mRNA expression of CyclinD1 and upregulated the mRNA expression of P53 in a concentration-dependent manner. Similarly, in the Western blot test, Figures 8C–E and Figures 9B–D showed that the protein level of P53 and Bax was significantly increased (*p <0.05), while the protein level of CyclinD1, CDK4, and Bcl-2 was significantly decreased (*p < 0.05) after SBP-2A treatment (Figures 10A–C). This phenomenon was consistent with the results obtained from the cell cycle assay. These data collectively indicated that SBP-2A blocked the DNA synthesis cycle in G1 phase by regulating the expression levels of P53 and CyclinD1 and induced apoptosis of HepG2 cells.

### Antitumor Activity of SBP-2A *in vivo*

The antitumour efficacy of different SBP-2A doses was evaluated in H22 tumour-bearing mice (Figure 10D). Figure 9A showed that the tumour weights in the groups treated with different doses of SBP-2A were smaller than those in the tumour model group. (25 mg/kg SBP-2A compared with the model group, ^*^ *p* < 0.05; 50 mg/kg SBP-2A compared with the model group, ^****^ *p* < 0.0001; 100 mg/kg SBP-2A compared with the model group, ^**^ *p* < 0.01). Furthermore, compared with the model group, the average inhibition rate of SBP-2A in doses of 50 mg/kg on tumor growth in mice was 40.33%, which significant inhibited the tumor growth of H22 tumor-bearing mice (Table 5).

**TABLE 5:** Tumour inhibition rate (%) of SBP-2A in H22 mice (*n* = 6).

Group	Dose (mg/kg)	Tumour weight (g)	Inhibition Rate (%)
Model	—	1.27 ± 0.09	—
SBP-2A	25	1.09 ± 0.16^*^	14.09
SBP-2A	50	0.76 ± 0.11^****^	40.33
SBP-2A	100	1.02 ± 0.11^**^	19.93
APS	100	0.86 ± 0.11^****^	32.63

Note: Mean ± SD, represent data, ^*^ *p* < 0.05, ^**^ *p* < 0.01, ^****^ *p* < 0.0001 vs. the model group.

## Discussions

The common design methods for the optimization of polysaccharide extraction include orthogonal tests and RSM. In the optimization of the SBP extraction process, previous studies used orthogonal testing. In this study, RSM was used for the first time to optimize the SBP extraction process. Compared with the orthogonal test, the RSM eliminated random errors in the experiment and allowed continuously analysis of the levels of the experiment during the optimization process to produce a continuous predictive model. In the single-factor experimental design, the polysaccharide extraction rate was lower at 100°C than at 90°C; this may have been due to the destruction of the polysaccharide structure when the temperature was too high (Zhang et al., 2015; Li et al., 2016; Cai et al., 2019). To facilitate practical operation, the process was improved as follows: the material–liquid ratio was 1:25, the extraction time was 2 h, and the extraction temperature was 90°C. Under these conditions, three groups of repeated validation experiments were carried out, and the SBP average extraction rate was 3.851 ± 0.13%, which was close to the predicted value of 4.06%. The result indicated that the model had good predictability and that the result of the RSM was reliable.

Five polysaccharide components were purified by ion column chromatography, and homogeneous acidic polysaccharide components with high purity, namely, SBP-1A and SBP-2A, were retained due to limitations of the experimental conditions. The molecular weight, monosaccharide composition and characteristics of SBP-1A and SBP-2A were preliminarily identified. The results showed that the molecular weight of SBP-1A (1.15 × 10^5^ Da) was less than that of SBP-2A (1.4 × 10^5^ Da), but both of them were medium-molecular-weight polysaccharides; these molecular weights were greater than that previously found for SBP. FT-IR analysis was used for rapid analysis of polysaccharides and to accurately identify specific absorption peaks (Vogt et al., 2019). SBP-1A and SBP-2A had a C=O stretching vibration at 1,634 cm^−1^ and contained uronic acid were proved, which is consistent with the monosaccharide composition of SBP determined by IC. In this study, comparing the anti-hepatoma activities of SBP, SBP-1A, and SBP-2A by MTT assay. The IC_50_ values for the 48 h inhibitory effect of SBP, SBP-1A, and SBP-2A on HepG2 cells were 1223, 891.7, and 548.3 μg/ml, respectively. It is worth noting that the IC_50_ value of SBP-2A was lower than that of SBP-1A, suggesting that SBP-2A may be more effective than SBP-1A in antitumour activity. Therefore, SBP-2A has high potential for application in cancer treatment research. The ability of the three polysaccharides from *Scutellaria barbata* to inhibit HepG2 cell proliferation was in order SBP-2A > SBP-1A > SBP. SBP-2A showed the strongest anti-hepatoma activity, which may be related to its high uronic acid content and the molecular weight of the polysaccharide molecules. The arabinose and galactose contents of SBP-2A are higher than those of SBP-1A. That is to say, the higher content of arabinose and galactose in the polysaccharide was, the stronger its antitumour effect. In addition, the C-H variable angle vibration occured near 1,380 cm^−1^, causing the characteristic absorption peak of *β*-glucan to appear. The transmittance of SBP-2A was significantly lower than that of SBP-1A. These results indicate that the *β*-glucan functional group of SBP-2A was stronger than that of SBP-1A, possibly leading to the difference in anti-hepatoma activity between these polysaccharides.

Many studies have shown that the low-toxicity natural polysaccharides extracted from Chinese herbal medicines inhibit the proliferation of tumour cells and selectively induce apoptosis (Sohretoglu et al., 2019). Different biological macromolecules can be extracted from different raw materials, and the types and structural characteristics of polysaccharides affect their biological activities (Zhao et al., 2017; Chen et al., 2020; Liu et al., 2020). It has been shown that long-term *in vitro* culturing or drug treatment of cells can inhibit cell colony formation (Lee et al., 2018; Chen et al., 2019). Therefore, we further verified the effect of SBP-2A on the inhibition of HepG2 cell proliferation with colony formation and Edu assays. *In vitro*, experiments preliminarily confirmed that SBP-2A inhibited the growth of HepG2 cells. We evaluated morphological changes of cells to determine the induced apoptosis response to drugs (Zhang et al., 2010; Drefs et al., 2017; Zhang W. et al., 2019). The morphological characteristics of HepG2 cells treated with SBP-2A for 48 h were directly observed by inverted microscopy. In this study, Hoechst 33258 staining confirmed that SBP-2A induced HepG2 cell apoptosis. To further evaluate the effect of SBP-2A on HepG2 cells, the DNA content was measured quantitatively after treatment with SBP-2A for 48 h by flow cytometry. In addition, the growth of normal and tumor cells was orderly in different stages of the cell cycle (Teloni et al., 2019), but cell cycle arrest and apoptosis can lead to programmed cell death (Zhang et al., 2017). We observed that SBP-2A induced apoptosis of HepG2 cells and arrested them in the G1 phase of the cell cycle. Cell cycle regulation is an important factor in tumorigenesis and development. There are many related molecules involved in the regulation of the G1 phase of the cell cycle, such as molecules belonging to the cyclin family and CDK family. CyclinD1 is the leading element in cell cycle regulation in the G1 phase; it promotes the transition from the G1 phase to S phase during the DNA synthesis cycle. The upregulation of CyclinD1 expression can induce tumour cell proliferation and lead to cancer characteristics (Choi et al., 2012). As a tumor suppressor, P53 is a key protein, which involved in DNA repair and apoptosis of damaged DNA cells. What’s more, CyclinD1 has a certain synergistic effect with P53. When CyclinD1 is overexpressed, it can combine with intracellular inactivated P53, to promote the infinite proliferation and continuous deterioration of cells (Sauter et al., 2002). The expression of CyclinD1 were downregulated and that of P53 were upregulated when HepG2 cells were treated with SBP-2A is the fingings confirmed that the combination of wild-type P53 and antisense CyclinD1 in HepG2 cells could improve the ability of SBP to induce tumour cell apoptosis and block the DNA synthesis cycle in the G0/G1 phase, this is mainly achieved by downregulating the protein expression of CDK and upregulating the Bax/Bcl-2 ratio. *In vivo*, in contrast to APS, SBP-2A at a dose of 50 mg/kg significantly inhibited the tumour growth in H22 tumour-bearing mice. It is essential to further clarify the components of SBP and its molecular targets that play a role in the drug effects. This could provide a basis for determining the effects of *Scutellaria barbata* polysaccharides anti-hepatoma and support their potential use as a functional medicinal component.

## Conclusion

In summary, the parameters for SBP extraction were optimized by RSM, and two homogeneous acidic polysaccharides with high purity were purified, SBP-1A and SBP-2A. The molecular weight, monosaccharide composition and characteristics of SBP-1A and SBP-2A were preliminarily identified. SBP, SBP-1A, and SBP-2A could inhibit the proliferation of HepG2 cells, but the ability of SBP-2A to inhibit the proliferation of HepG2 cells was better than that of SBP and SBP-1A. Moreover, SBP-2A could change the morphology of HepG2 cells and significantly induce apoptosis, which may be achieved by upregulating the expression of p53, Bax/Bcl-2 ratio and downregulating the expression of CyclinD1 and CDK4, to block HepG2 cells in the G0/G1 phase of the cell cycle. These results showed that SBP-2A isolated and purified from *Scutellaria barbata* showed antitumour activity *in vivo* and *in vitro*, which is worthy of further evaluation. This study, through in-depth exploration, provides information on the molecular mechanism of the anti-hepatoma effect of SBP.

## Consent to Publish

All authors reviewed the results and approved the final version of the manuscript.

## Data Availability

The raw data supporting the conclusions of this article will be made available by the authors, without undue reservation.

## Abbreviations

SBP, Crude Polysaccharide of *Scutellaria Barbata*; NEAAs, Non-Essential Amino Acids; FBS, Fetal Bovine Serum; MTT, 3-(4,5-Dimethylthiazol-2-yl)-2,5-diphenyltetrazolium bromide; FT-IR, Fourier Transform Infrared; CV, Coefficient of Variation; HPLC, High-Performance Liquid Chromatography.

## References

[B1] Bargougui K. Athmouni K. Chaieb M. (2019). Optimization, Characterization and Hepatoprotective Effect of Polysaccharides Isolated from Stipa Parviflora Desf. Against CCl4 Induced Liver Injury in Rats Using Surface Response Methodology (RSM). Int. J. Biol. Macromol 132, 524–533. 10.1016/j.ijbiomac.2019.03.216 30936007

[B2] Cai L. Chen B. Yi F. Zou S. (2019). Optimization of Extraction of Polysaccharide from Dandelion Root by Response Surface Methodology: Structural Characterization and Antioxidant Activity. Int. J. Biol. Macromol 140, 907–919. 10.1016/j.ijbiomac.2019.08.161 31437509

[B3] Cao P. Wu S. Wu T. Deng Y. Zhang Q. Wang K. (2020). The Important Role of Polysaccharides from a Traditional Chinese Medicine-Lung Cleansing and Detoxifying Decoction against the COVID-19 Pandemic. Carbohydr. Polym. 240, 116346. 10.1016/j.carbpol.2020.116346 32475597 PMC7175912

[B4] Chen H. Huang X. Fu C. Wu X. Peng Y. Lin X. (2019). Recombinant Klotho Protects Human Periodontal Ligament Stem Cells by Regulating Mitochondrial Function and the Antioxidant System during H2O2-Induced Oxidative Stress. Oxid Med. Cel Longev 2019, 9261565. 10.1155/2019/9261565 PMC6914990 31885825

[B5] Chen J. Y. Sun X. Y. Ouyang J. M. (2020). Modulation of Calcium Oxalate Crystal Growth and Protection from Oxidatively Damaged Renal Epithelial Cells of Corn Silk Polysaccharides with Different Molecular Weights. Oxid Med. Cel Longev 2020, 6982948. 10.1155/2020/6982948 PMC7008244 32089775

[B6] Chen Y. Y. Xue Y. T. (2019). Optimization of Microwave Assisted Extraction, Chemical Characterization and Antitumor Activities of Polysaccharides from Porphyra Haitanensis. Carbohydr. Polym. 206, 179–186. 10.1016/j.carbpol.2018.10.093 30553311

[B7] Choi Y. J. Li X. Hydbring P. Sanda T. Stefano J. Christie A. L. (2012). The Requirement for Cyclin D Function in Tumor Maintenance. Cancer Cell 22 (4), 438–451. 10.1016/j.ccr.2012.09.015 23079655 PMC3487466

[B8] Chylińska M. Szymańska-Chargot M. Zdunek A. (2016). FT-IR and FT-Raman Characterization of Non-cellulosic Polysaccharides Fractions Isolated from Plant Cell wall. Carbohydr. Polym. 154, 48–54. 10.1016/j.carbpol.2016.07.121 27577895

[B9] Drefs M. Thomas M. N. Guba M. Angele M. K. Werner J. Conrad M. (2017). Modulation of Glutathione Hemostasis by Inhibition of 12/15-Lipoxygenase Prevents ROS-Mediated Cell Death after Hepatic Ischemia and Reperfusion. Oxid Med. Cel Longev 2017, 8325754. 10.1155/2017/8325754 PMC5546123 28811867

[B10] Dubois M. Gilles K. Hamilton J. K. Rebers P. A. Smith F. (1951). A Colorimetric Method for the Determination of Sugars. Nature 168 (4265), 167. 10.1038/168167a0 14875032

[B11] Finn R. S. Ikeda M. Zhu A. X. Sung M. W. Baron A. D. Kudo M. (2020). Phase Ib Study of Lenvatinib Plus Pembrolizumab in Patients with Unresectable Hepatocellular Carcinoma. J. Clin. Oncol. 38 (26), 2960–2970. 10.1200/jco.20.00808 32716739 PMC7479760

[B12] Gan Q. X. Wang J. Hu J. Lou G. H. Xiong H. J. Peng C. Y. (2020). Modulation of Apoptosis by Plant Polysaccharides for Exerting Anti-cancer Effects: A Review. Front. Pharmacol. 11, 792. 10.3389/fphar.2020.00792 32536869 PMC7267062

[B13] Hui Y. Jun-Li H. Chuang W. (2019). Anti-oxidation and Anti-aging Activity of Polysaccharide from Malus Micromalus Makino Fruit Wine. Int. J. Biol. Macromol 121, 1203–1212. 10.1016/j.ijbiomac.2018.10.096 30342941

[B14] Jia X. Ding C. Yuan S. Zhang Z. Chen Y. Du L. (2014). Extraction, Purification and Characterization of Polysaccharides from Hawk tea. Carbohydr. Polym. 99, 319–324. 10.1016/j.carbpol.2013.07.090 24274513

[B15] Lee H. Jang Y. Park S. Jang H. Park E. J. Kim H. J. (2018). Development and Evaluation of a CEACAM6-Targeting Theranostic Nanomedicine for Photoacoustic-Based Diagnosis and Chemotherapy of Metastatic Cancer. Theranostics 8 (15), 4247–4261. 10.7150/thno.25131 30128051 PMC6096393

[B16] Li F. Gao J. Xue F. Yu X. Shao T. (2016). Extraction Optimization, Purification and Physicochemical Properties of Polysaccharides from Gynura Medica. Molecules 21 (4), 397. 10.3390/molecules21040397 27023496 PMC6273717

[B17] Li H. Su J. Jiang J. Li Y. Gan Z. Ding Y. (2019a). Characterization of Polysaccharide from Scutellaria Barbata and its Antagonistic Effect on the Migration and Invasion of HT-29 Colorectal Cancer Cells Induced by TGF-Β1. Int. J. Biol. Macromol 131, 886–895. 10.1016/j.ijbiomac.2019.03.053 30857966

[B18] Li K. Cui L. J. Cao Y. X. Li S. Y. Shi L. X. Qin X. M. (2020). UHPLC Q-Exactive MS-Based Serum Metabolomics to Explore the Effect Mechanisms of Immunological Activity of Astragalus Polysaccharides with Different Molecular Weights. Front. Pharmacol. 11, 595692. 10.3389/fphar.2020.595692 33390982 PMC7774101

[B19] Li L. Xu X. Wu L. Zhu H. He Z. Zhang B. (2019b). Scutellaria Barbata Polysaccharides Inhibit Tumor Growth and Affect the Serum Proteomic Profiling of Hepatoma H22-bearing M-ice. Mol. Med. Rep. 19 (3), 2254–2262. 10.3892/mmr.2019.9862 30664217 PMC6390040

[B20] Li W. Xiao H. (2021). Scutellaria Barbata D. Don Polysaccharides Inhibit High Glucose-Induced Proliferation and Angiogenesis of Retinal Vascular Endothelial Cells. Diabetes Metab. Syndr. Obes. 14, 2431–2440. 10.2147/dmso.S296164 34103952 PMC8180288

[B21] Liu H. Sun X. Y. Wang F. X. Ouyang J. M. (2020). Regulation on Calcium Oxalate Crystallization and Protection on HK-2 Cells of Tea Polysaccharides with Different Molecular Weights. Oxid Med. Cel Longev 2020, 5057123. 10.1155/2020/5057123 PMC7243009 32454940

[B22] Ma T. Sun X. Tian C. Luo J. Zheng C. Zhan J. (2016). Polysaccharide Extraction from Sphallerocarpus Gracilis Roots by Response Surface Methodology. Int. J. Biol. Macromol 88, 162–170. 10.1016/j.ijbiomac.2016.03.058 27032488

[B23] Mkadmini Hammi K. Hammami M. Rihouey C. Le Cerf D. Ksouri R. Majdoub H. (2016). Optimization Extraction of Polysaccharide from Tunisian Zizyphus lotus Fruit by Response Surface Methodology: Composition and Antioxidant Activity. Food Chem. 212, 476–484. 10.1016/j.foodchem.2016.06.004 27374558

[B24] Qu J. Huang P. Zhang L. Qiu Y. Qi H. Leng A. (2020). Hepatoprotective Effect of Plant Polysaccharides from Natural Resources: A Review of the Mechanisms and Structure-Activity Relationship. Int. J. Biol. Macromol 161, 24–34. 10.1016/j.ijbiomac.2020.05.196 32485257

[B25] Qu Y. Li C. Zhang C. Zeng R. Fu C. (2016). Optimization of Infrared-Assisted Extraction of Bletilla Striata Polysaccharides Based on Response Surface Methodology and Their Antioxidant Activities. Carbohydr. Polym. 148, 345–353. 10.1016/j.carbpol.2016.04.081 27185148

[B26] Sauter E. R. Takemoto R. Litwin S. Herlyn M. (2002). p53 Alone or in Combination with Antisense Cyclin D1 Induces Apoptosis and Reduces Tumor Size in Human Melanoma. Cancer Gene Ther. 9 (10), 807–812. 10.1038/sj.cgt.7700492 12224020

[B27] Singal A. G. Lampertico P. Nahon P. (2020). Epidemiology and Surveillance for Hepatocellular Carcinoma: New Trends. J. Hepatol. 72 (2), 250–261. 10.1016/j.jhep.2019.08.025 31954490 PMC6986771

[B28] Sohretoglu D. Zhang C. Luo J. Huang S. (2019). ReishiMax Inhibits mTORC1/2 by Activating AMPK and Inhibiting IGFR/PI3K/Rheb in Tumor Cells. Signal. Transduct Target. Ther. 4, 21. 10.1038/s41392-019-0056-7 31637001 PMC6799808

[B29] Sun P. Sun D. Wang X. (2017). Effects of Scutellaria Barbata Polysaccharide on the Proliferation, Apoptosis and EMT of Human colon Cancer HT29 Cells. Carbohydr. Polym. 167, 90–96. 10.1016/j.carbpol.2017.03.022 28433181

[B30] Tabrizian P. Jibara G. Shrager B. Schwartz M. Roayaie S. (2015). Recurrence of Hepatocellular Cancer after Resection: Patterns, Treatments, and Prognosis. Ann. Surg. 261 (5), 947–955. 10.1097/sla.0000000000000710 25010665

[B31] Teloni F. Michelena J. Lezaja A. Kilic S. Ambrosi C. Menon S. (2019). Efficient Pre-mRNA Cleavage Prevents Replication-Stress-Associated Genome Instability. Mol. Cel 73 (4), 670–e12. 10.1016/j.molcel.2018.11.036 PMC6395949 30639241

[B32] Vogt S. Löffler K. Dinkelacker A. G. Bader B. Autenrieth I. B. Peter S. (2019). Fourier-Transform Infrared (FTIR) Spectroscopy for Typing of Clinical *Enterobacter cloacae* Complex Isolates. Front. Microbiol. 10, 2582. 10.3389/fmicb.2019.02582 31781074 PMC6851243

[B33] Wang D. Liu Y. Zhao W. (2021). The Adjuvant Effects on Vaccine and the Immunomodulatory Mechanisms of Polysaccharides from Traditional Chinese Medicine. Front. Mol. Biosci. 8, 655570. 10.3389/fmolb.2021.655570 33869288 PMC8047473

[B34] Wang R. Chen P. Jia F. Tang J. Ma F. (2012). Optimization of Polysaccharides from Panax Japonicus C.A. Meyer by RSM and its Anti-oxidant Activity. Int. J. Biol. Macromol 50 (2), 331–336. 10.1016/j.ijbiomac.2011.12.023 22214823

[B35] Wei D. Chen T. Yan M. Zhao W. Li F. Cheng W. (2015). Synthesis, Characterization, Antioxidant Activity and Neuroprotective Effects of Selenium Polysaccharide from Radix Hedysari. Carbohydr. Polym. 125, 161–168. 10.1016/j.carbpol.2015.02.029 25857971

[B36] Wu H. Li M. Yang X. Wei Q. Sun L. Zhao J. (2020). Extraction Optimization, Physicochemical Properties and Antioxidant and Hypoglycemic Activities of Polysaccharides from Roxburgh Rose (Rosa Roxburghii Tratt.) Leaves. Int. J. Biol. Macromol 165 (Pt A), 517–529. 10.1016/j.ijbiomac.2020.09.198 33002536

[B37] Wu Y. Chen D. F. (2009). Anti-complementary Effect of Polysaccharide B3-PS1 in Herba Scutellariae Barbatae (Scutellaria Barbata). Immunopharmacol Immunotoxicol 31 (4), 696–701. 10.3109/08923970903095314 19874244

[B38] Xie M. Tao W. Wu F. Wu K. Huang X. Ling G. (2021). Anti-hypertensive and Cardioprotective Activities of Traditional Chinese Medicine-Derived Polysaccharides: A Review. Int. J. Biol. Macromol 185, 917–934. 10.1016/j.ijbiomac.2021.07.008 34229020

[B39] Yang J. Yang G. Hou G. Liu Q. Hu W. Zhao P. U. (2015). Scutellaria Barbata D. Don Polysaccharides Inhibit the Growth of Calu-3 Xenograft Tumors via Suppression of the HER2 Pathway and Angiogenesis. Oncol. Lett. 9 (6), 2721–2725. 10.3892/ol.2015.3127 26137135 PMC4473715

[B40] Yang J. D. Hainaut P. Gores G. J. Amadou A. Plymoth A. Roberts L. R. (2019). A Global View of Hepatocellular Carcinoma: Trends, Risk, Prevention and Management. Nat. Rev. Gastroenterol. Hepatol. 16 (10), 589–604. 10.1038/s41575-019-0186-y 31439937 PMC6813818

[B41] Yang X. Yang Y. Tang S. Tang H. Yang G. Xu Q. (2014). Anti-tumor Effect of Polysaccharides from Scutellaria Barbata D. Don on the 95-D Xenograft Model via Inhibition of the C-Met Pathway. J. Pharmacol. Sci. 125 (3), 255–263. 10.1254/jphs.13276fp 25048016

[B42] Yao Y. L. Shu C. Feng G. Wang Q. Yan Y. Y. Yi Y. (2020). Polysaccharides from Pyracantha Fortuneana and its Biological Activity. Int. J. Biol. Macromol 150, 1162–1174. 10.1016/j.ijbiomac.2019.10.125 31794823

[B43] Ye C. L. Huang Q. (2012). Extraction of Polysaccharides from Herbal Scutellaria Barbata D. Don (Ban-Zhi-Lian) and Their Antioxidant Activity. Carbohydr. Polym. 89 (4), 1131–1137. 10.1016/j.carbpol.2012.03.084 24750924

[B44] Yeon E. T. Lee J. W. Lee C. Jin Q. Jang H. Lee D. (2015). Neo-Clerodane Diterpenoids from Scutellaria Barbata and Their Inhibitory Effects on LPS-Induced Nitric Oxide Production. J. Nat. Prod. 78 (9), 2292–2296. 10.1021/acs.jnatprod.5b00126 26331882

[B45] Yuan Q. Yuan Y. Zheng Y. Sheng R. Liu L. Xie F. (2021). Anti-cerebral Ischemia Reperfusion Injury of Polysaccharides: A Review of the Mechanisms. Biomed. Pharmacother. 137, 111303. 10.1016/j.biopha.2021.111303 33517189

[B46] Zhang C. Zhao F. Li R. Wu Y. Liu S. Liang Q. (2019a). Purification, Characterization, Antioxidant and Moisture-Preserving Activities of Polysaccharides from Rosa Rugosa Petals. Int. J. Biol. Macromol 124, 938–945. 10.1016/j.ijbiomac.2018.11.275 30503792

[B47] Zhang F. Shi J. J. Thakur K. Hu F. Zhang J. G. Wei Z. J. (2017). Anti-Cancerous Potential of Polysaccharide Fractions Extracted from Peony Seed Dreg on Various Human Cancer Cell Lines via Cell Cycle Arrest and Apoptosis. Front. Pharmacol. 8, 102. 10.3389/fphar.2017.00102 28316571 PMC5334287

[B48] Zhang T. T. Lu C. L. Jiang J. G. Wang M. Wang D. M. Zhu W. (2015). Bioactivities and Extraction Optimization of Crude Polysaccharides from the Fruits and Leaves of Rubus Chingii Hu. Carbohydr. Polym. 130, 307–315. 10.1016/j.carbpol.2015.05.012 26076631

[B49] Zhang W. Lei Z. Meng J. Li G. Zhang Y. He J. (2019b). Water Extract of Sporoderm-Broken Spores of Ganoderma Lucidum Induces Osteosarcoma Apoptosis and Restricts Autophagic Flux. Onco Targets Ther. 12, 11651–11665. 10.2147/ott.S226850 32021244 PMC6942530

[B50] Zhang W. L. Zhu L. Jiang J. G. (2014). Active Ingredients from Natural Botanicals in the Treatment of Obesity. Obes. Rev. 15 (12), 957–967. 10.1111/obr.12228 25417736

[B51] Zhang Y. Soto J. Park K. Viswanath G. Kuwada S. Abel E. D. (2010). Nuclear Receptor SHP, a Death Receptor that Targets Mitochondria, Induces Apoptosis and Inhibits Tumor Growth. Mol. Cel Biol 30 (6), 1341–1356. 10.1128/mcb.01076-09 PMC2832505 20065042

[B52] Zhao L. Wang Y. Shen H. L. Shen X. D. Nie Y. Wang Y. (2012). Structural Characterization and Radioprotection of Bone Marrow Hematopoiesis of Two Novel Polysaccharides from the Root of Angelica Sinensis (Oliv.) Diels. Fitoterapia 83 (8), 1712–1720. 10.1016/j.fitote.2012.09.029 23063893

[B53] Zhao X. Li J. Liu Y. Wu D. Cai P. Pan Y. (2017). Structural Characterization and Immunomodulatory Activity of a Water Soluble Polysaccharide Isolated from Botrychium Ternatum. Carbohydr. Polym. 171, 136–142. 10.1016/j.carbpol.2017.05.014 28578947

[B54] Zhao Z. Y. Zhang Q. Li Y. F. Dong L. L. Liu S. L. (2015). Optimization of Ultrasound Extraction of Alisma Orientalis Polysaccharides by Response Surface Methodology and Their Antioxidant Activities. Carbohydr. Polym. 119, 101–109. 10.1016/j.carbpol.2014.11.052 25563949

